# Early to Mid-Holocene Palaeoenvironment Change and Sedimentary Evolution in the Xianghu Area, Zhejiang

**DOI:** 10.3390/ijerph17197099

**Published:** 2020-09-28

**Authors:** Longbin Sha, Xianfu Li, Jiabing Tang, Junwu Shu, Weiming Wang, Dongling Li

**Affiliations:** 1 Department of Geography and Spatial Information Techniques, Ningbo University, Ningbo 315211, China; shalongbin@nbu.edu.cn (L.S.); xianfuli99@163.com (X.L.); jiabingtang123@163.com (J.T.); 2 Institute of East China Sea, Ningbo University, Ningbo 315211, China; 3 State Key Laboratory of Estuarine and Coastal Research, East China Normal University, Shanghai 200062, China; 4 State Key Laboratory of Palaeobiology and Stratigraphy, Nanjing Institute of Geology and Palaeontology, CAS, Nanjing 210008, China; junwushu@126.com (J.S.); wmwang@nigpas.ac.cn (W.W.)

**Keywords:** total organic carbon, stable organic carbon isotope, paleoclimatic environment, Xianghu area

## Abstract

A 2.5 m long sediment core (XH-2) obtained from Xianghu area, near the Kuahuqiao site, were analyzed for grain size, diatom index, and geochemistry of organic carbon. The results of the total organic carbon (TOC) and stable organic carbon isotope (δ^13^C) in sediment samples from core XH-2 in the Xianghu area in Zhejiang Province have revealed the evolution history of sedimentary environmental and climatic changes during the breeding–prosperity–decline period of the Kuahuqiao culture. During 9300–8200 cal a BP, TOC contents were relatively high and stable, whereas δ^13^C values tended to be negative. This condition indicated that the climate was humid, and the sedimentary environment in the Xianghu area was stable. During 8200–7500 cal a BP, TOC contents presented a fluctuating declining trend, and δ^13^C values were significantly high, implying that the climate was arid, and the Xianghu area was gradually reduced to land. Thus, conducive conditions were provided for the development of the Kuohuqiao culture (7700–7400 cal a BP). From 7500 cal a BP, TOC contents obviously declined, and δ^13^C values were partially low, suggesting strengthened hydrodynamic force and wet conditions in the Xianghu area. This condition was related to the rise in sea level at approximately 7400 cal a BP, and the Kuahuqiao site became obsolete due to the transgression event. The TOC contents in core XH-2 were remarkably influenced by grain size, whereas no significant correlation existed between the δ^13^C variability and grain size. Sedimentary environment changes in the Xianghu area from 9300 to 6600 cal a BP, which was reflected by the TOC and δ^13^C records in core XH-2, accorded with the diatom results in this core and those in the Baima Lake area.

## 1. Introduction

Coastal and delta areas are highly crucial because they are transitional zones from land to sea and centres for social, economic and cultural development [1]. Neolithic cultures developed and flourished in the Yangtze Delta [2,3,4,5,6]. Many elaborate cultures dependent on hunting-gathering or farming emerged in the coastal areas of the world during mid-Holocene, when the sea-level rise decelerated and coastal stabilisation provided exploitable land surfaces and coastal and estuarine resources [3,6,7,8,9] However, the depositional environment and human activity in the Yangtze Delta in the early Neolithic predating 7500 cal a BP remains relatively unknown.

A stable organic carbon isotope (δ^13^C) is a crucial index for the source inversion of organic matters. The principle of this index is that organic matters from different sources utilise CO_2_ through various means, resulting in δ^13^C fractionation. Studies have demonstrated that the terrestrial organic matter exhibits lower δ^13^C values (between −32‰ and −21‰) than the marine organic matters due to the predominant contribution of C3 plant detritus [10,11,12]. δ^13^C has been used successfully to distinguish the provenance of organic materials in coastal and marine sediments and to reveal palaeoenvironmental and sea-level changes [13,14,15,16,17,18,19,20]. Additionally, δ^13^C, which can reflect humidity variations occurring in the study area, is a vital index for palaeoenvironmental changes. Wang [21] studied the surface sediment δ^13^C in 55 lakes on the Inner Mongolian Plateau and in its adjacent areas. The δ^13^C presented an extremely strong negative correlation with the precipitation. The corresponding δ^13^C value becomes partially negative with an increase in the precipitation.

Diatoms are microscopic algae found virtually everywhere in the presence of water, and each habitat produces its own characteristic diatom flora depending on the chemical and physical environment [22]. Diatom cell walls which are ornamented with intricate, taxonomically diagnostic patterns, usually preserve well in the sediments and thus provide an indicator of the temporal and spatial trends in environmental changes [23,24]. Therefore, diatom fossils in the sediments are excellent interpretative tools for understanding past environmental conditions [23,24,25].

This paper focuses on: (1) the source of organic matter in the Xianghu area, (2) correlations between the geochemical index and diatom record, and (3) interpretation of palaeogeographic and palaeoclimatic changes during Holocene in the Xianghu area.

## 2. Study Area

The Ningshao plain is located in the northeast of Zhejiang Province and is a long and narrow east–west coastal plain. Modern climate in the study area is typically monsoonal with an annual temperature of 16.1 °C and an annual precipitation of 1402.5 mm, most of which falls in the summer [5]. This plain reaches the East China Sea coast in the east with an intense land-sea interaction and comprises the Ningbo, Sanbei, Shangyu, Nansha, and Xiaoshao plains. Starting from Xiaoshan in the west, it lies close to three major mountain ranges, namely the Siming, Tiantai, and Kuaiji mountains. In the south, it extends to the south bank of the Qiantang river and outstretches to the Hangjianghu plain across the Qiantang river. The Xianghu lake is located on the Ningshao plain with a complex surrounding topography. Rivers cross the lake [26], and its west, north, south, and east sides are encircled by three rivers, namely the Qiantang, Puyang, and Xixiao rivers [27]. It is a sensitive area responding to global climatic and environmental changes that lead to sea-level fluctuations [28,29].

The palaeoenvironment and sea level of the Ningshao plain underwent some crucial changes, which influenced the rise, fall, and development of several Neolithic civilizations such as Kuahuqiao (8000–7000 cal a BP) [4] and Hemudu (5000–3000 cal a BP). For example, the Kuahuqiao culture near the Xianghu area declined because of transgression approximately 7500 years ago; thus, the rise and fall of civilizations are closely related to sea level fluctuations and the resulting environmental changes [30]. Therefore, the Ningshao plain is ideal for determining whether the effects of climate changes on human settlements are reflected in studies from lake sediments, specifically total organic carbon (TOC), δ^13^C, and grain size.

## 3. Materials and Methods

### 3.1. TOC and δ^13^C Tests

**Figure 1:**
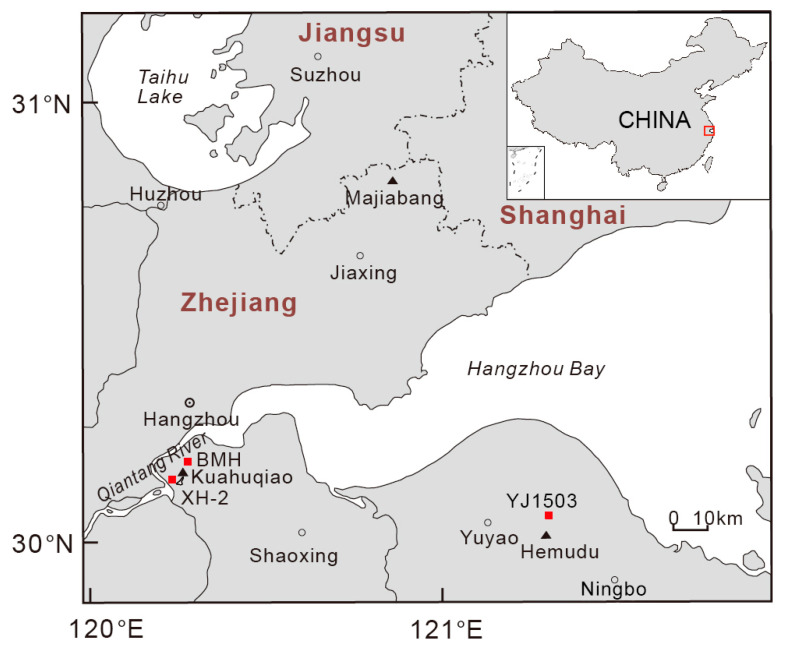
Location map with the core XH-2 site in the Xianghu area, Zhejiang province, eastern China.

Core XH-2 (30°07′17″ N, 120°11′38″ E) is located in the west of the Ningshao plain and southwest of the Kuahuqiao Neolithic cultural site (30°08′42″ N, 120°13′02″ E) (Figure 1). It was drilled by the Nanjing Institute of Geology and Palaeontology, the Chinese Academy of Sciences, and its ground elevation was approximately 4 m. Artificial soil was conducted at 0–100 cm of core XH-2. Thus, 113 sediment samples at 131–2491 cm were selected for analyzing TOC and δ^13^C (Table 1).

A vacuum freezing and drying sample (about 0.5 g) which was screened by an 80-mesh sieve was put into a centrifuging tube, in which 5.0 mL 10% HCl was added. The mixture was stirred continuously. Then, deionized water was added into the centrifuging tube for cleaning and centrifuging process. This process was repeated until the pH of supernatant liquid was close to 7, before the supernatant liquid was eliminated. The rest sample was dried overnight in an oven under 60 °C. About 50–60 mg of the sample was weighed by a balance and wrapped in a small stannum box. Then, the sample was crushed into sheets. TOC was measured by an element analyzer vario MACRO cube [30]. The detection limit of carbon was 8 × 10^6^ g, the precision of the instrument was <0.01%, and the recovery efficiency was >99.5% [31]. Numerical value of δ^13^C was measured by a DELTA plusXP stable isotope mass spectrometer (ThermoFinnigan, San Jose, CA, USA) [32]. Stable isotopic ratios of δ^13^C were determined as follows: δ^13^C = (R_sample_/R_standard_ − 1) × 1000, where R_sample_ is the test result of ratio ^13^C/^12^C, and R_standard_ is expressed relative to the Vienna Pee Dee Belemnite (V-PDB) standard. The analytical precision was ±0.2‰ for δ^13^C, based on the analysis of the standards [32].

### 3.2. Diatom Analysis

The diatom analysis in the present study used about 5–6 mg of freeze-dried particles from each sample. The samples were weighed with a precision of 0.1 mg. The permanent slides for chemical treatment and microscopic observation was prepared according to the improved method established by the Alfred Wegener Institute for Polar and Marine Research [33]. After addition of 10% hydrochloric acid (HCl) and 30% hydrogen peroxide (H_2_O_2_) to the sample, it was heated to remove lime and organic matter. After complete digestion, the mixture was carefully stirred with distilled water and kept for 24 h to precipitate the particles. Subsequently, the supernatant was siphoned off, and the washing process was repeated four times to remove excess HCl, H_2_O_2,_ and the reaction solution. The remaining samples were diluted to 20 mL, and then 2 mL of gelatin solution was added to the samples to accelerate the settling process [34]. The mixed sample solution was gently poured into the petri dish, and two 22 mm × 22 mm cover slides were pre-fixed in the petri dish. After 24 h of precipitation, an absorbent paper strip was used to remove the supernatant from the petri dish [34]. When the material was completely dried, the cover was transferred to the tagged carrier and installed with Naphrax (dn = 1.73). Diatoms were counted and identified through a Leica DM2500 phase contrast microscope under 1000 times magnification. Diatom flux was then calculated using the following formula:

(1) $$A=\frac{N\times S}{n\times a\times m}$$

where, *A* is the diatom abundance (valves/g), *N* is the number of diatoms counted under the microscope, *S* is the area of the petri dish, *n* is the number of fields of vision counted for diatoms under the microscope, *a* is the area of one field of vision, and *m* is the dry weight of sample (g) used for diatom analysis [34].

### 3.3. Grain Size Analysis

Grain size determinations were made on 113 samples using a Beckman Coulter laser diffraction particle size analyser (LS13320), with a measurement range of 0.04–2000 mm, at the State Key Laboratory of Estuarine and Coastal Research, East China Normal University. Samples were dried at 40 °C for 24 h, followed by the addition of 10% HCl to eliminate carbonates, 10% H_2_O_2_ to eliminate organic matter, and finally Na (PO3)_6_ to disperse the samples before testing.

### 3.4. Accelerator Mass Spectrometry (AMS) ^14^C Dating

**Figure 2:**
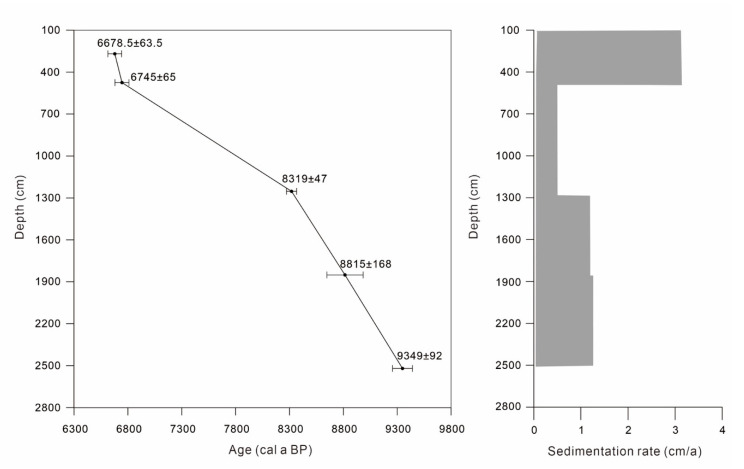
Age model and sedimentation rate based on AMS^14^C age determinations of core XH-2 [35].

Six samples of plant fragments and organic-rich sediment were collected from the core for accelerator mass spectrometry (AMS) ^14^C dating at Beta Analytic, FL, USA. All conventional ages were calibrated using Calib702 software (Table 2, Figure 2). The age of layer 595 cm was 13,060 cal a BP. The age-dating result was partially old and considerably deviated from the result in adjacent strata probably due to the sediment age deviation caused by geological processes, difference in age-dating materials, improper sample pretreatment process, or problem occurring in the age-dating process. Therefore, the age-dating result at layer 595 cm was excluded, and the depth-age model graph of core XH-2 was obtained. The linear interpolation method was adopted for age series within age control points, whereas the linear extrapolation method was adopted for those beyond the age control points for the calculation of ages at different depths. The sediments of core XH-2 in the Xianghu area during 9300–6600 cal a BP (2491–131 cm) were obtained in the end.

## 4. Results

### 4.1. Principal Component Analysis of Diatom Taxa

A total of 124 diatom species in 37 genera were identified from the analysis of core XH-2 sediment samples from Xianghu lake [35]. The fresh water species mainly comprised *Aulacoseira granulata*, *Achnanthes minutissima*, and *Achnanthes* spp. Brackish-water species comprised *Actinocyclus octonarius*, *Cyclotella striata*, *Actinoptychus senarius*, *Paralia sulcata*, *Diploneis smithii*, and *Nitzschia granulata*, whereas marine species comprised *Coscinodiscus argus*, *Coscinodiscus blandus*, *Coscinodiscus ellipticus*, *Coscinodiscus lacustris*, *Coscinodiscus radiatus*, *Thalassionema nitzschioides*, and *Nitzschia levidensis* (see Appendix A for taxonomic details).

**Figure 3:**
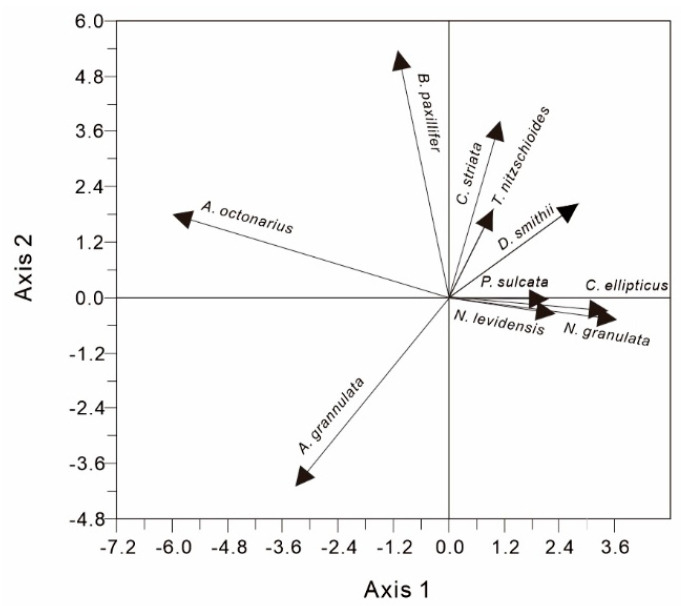
Principal component analysis (PCA) taxa scatter diagram of axis 1 against axis 2.

Statistically significant directions of variation within the samples were identified through a linear method of principal component analysis (PCA). In a PCA species plot, the species are represented by vectors and positioned based on their scores related to the ordination axes [4]. Thus, the correlation between the species and the axis may be used to estimate the environmental significance of the axis. Five characteristics of the axis values were 0.352, 0.161, 0.129, 0.084, and 0.061. The cumulative contribution rate of axis 1 and axis 2 to the total variance of all species variables reached 51.3%, whereas the remaining contribution rates were small. Thus, axis 1 and axis 2 can explain most of the data information (Figure 3).

**Figure 4:**
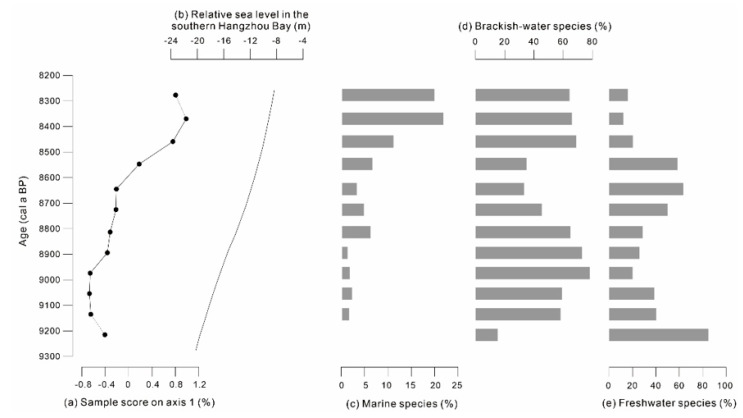
(**a**) Sample score on axis 1, (**b**) relative sea level in the southern Hangzhou Bay [36] and (**c**–**e**) abundance of marine diatoms, brackish-water diatom species, and freshwater species [35].

Abundance of marine species (*N. levidensis*, *C. ellipticus*, and *T. nitzschioides*) was positively correlated with axis 1 (Figure 4a,c), and the Pearson correlation coefficient between them was 0.89 (*p* < 0.01). Brackish-water species such as *C. striata*, *D. smithii*, and *N. granulata* were distributed on the right side of the principal component load diagram, exhibiting an obviously positive correlation with axis 1 (Figure 3). Additionally, the freshwater species *A. granulata* was distributed on the left of the principal component load diagram, exhibiting significant negative correlation with axis 1. Therefore, sample score on axis 1 can be used to indicate the influence of sea water on the Xianghu area, and a high score on axis 1 corresponds to the strengthened influence of sea water. Furthermore, a gradual increase in sample score on axis 1 between 9300 and 8200 cal a BP was in good agreement with sea level rise in the southern Hangzhou Bay estimated by Liu et al. [36] (Figure 4b). Thus, it is reliable to use the sample score on axis 1 to indicate changes in marine influence on the Xianghu area.

### 4.2. TOC Contents and δ^13^C of Core XH-2

**Figure 5:**
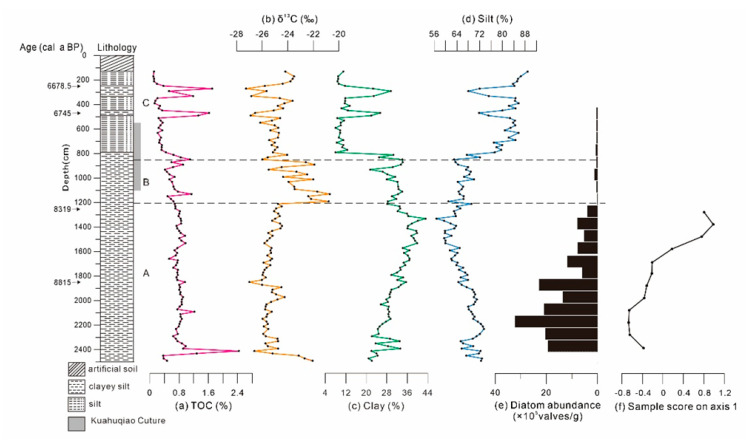
Results of (**a**) TOC content, (**b**) δ^13^C, (**c**) clay content, (**d**) silt content [35], (**e**) diatom abundance, and (**f**) Sample score on axis 1 of core XH-2.

The lithology was relatively homogenous, dominated by grey silt and clayey silt from top to bottom for core XH-2, with the mean of 3.3% sand (>63 μm), 70.5% silt (4–63 μm), and 26.2% clay (<4 μm) [35]. The TOC variation range of core XH-2 during 9300–6600 cal a BP was 0.10–2.44%, and the mean value was 0.682% (Figure 5a). The δ^13^C values of core XH-2 fluctuated by a large margin between approximately −27.26‰ and −20.71‰ during 9300–6600 cal a BP, and the mean value was −24.88‰. The maximum δ^13^C value of −20.71‰ appeared at 1131-cm depth (approximately 8050 cal a BP), whereas the minimum δ^13^C value of −27.26‰ appeared at 271 cm on the top of core XH-2 (approximately 6700 cal a BP) (Figure 5b).

The TOC contents and δ^13^C could be divided into the following three units (A–C) according to the variation characteristics:

Unit A was 2491–1231 cm (approximately 9300–8300 cal a BP), and the TOC contents fluctuated between 0.37% and 2.44%, which was higher than the mean value. The maximum value appeared in approximately 9250 cal a BP, although this is represented by only a single data point. Between 2491 and 1211 cm (approximately 9300–8200 cal a BP), the δ^13^C values fluctuated between −27.00‰ and −22.06‰ (mean value: −25.24‰). All δ^13^C values in this unit, with the exception of approximately 9300 cal a BP, were lower than the mean value.

Unit B was 1211–831 cm (approximately 8200–7450 cal a BP), and the TOC contents fluctuated between 0.41% and 1.14%. The TOC contents in this unit fluctuated considerably compared with those in the unit A. During approximately 8000–7600 cal a BP, the TOC contents were lower than the mean value. The δ^13^C values were positive compared with those in unit A and fluctuated from −25.49‰ to −20.71‰ (mean value: −22.84‰). All δ^13^C values in unit B, with the exception of approximately 7700 cal a BP, were remarkably higher than the mean value.

Unit C was 811–131 cm (approximately 7400–6600 cal a BP). The TOC contents ranged from 0.10% to 1.71%, declined obviously, and were lower than the mean value during the whole approximately 7400–6800 cal a BP compared with that in unit B. However, from 6800 cal a BP, the TOC contents fluctuated at a relatively high level. The δ^13^C values were evidently negative compared with that in unit B and fluctuated between −27.26‰ and −23.51‰ (mean value: −25.06‰). Most δ^13^C values in this unit, with the exception of approximately 6700 cal a BP, were lower than the mean value.

## 5. Discussion

### 5.1. 9300–8200 cal a BP

Piecewise calculation of the Pearson correlation coefficients of TOC with clay and silt contents was conducted to investigate the influencing factors of TOC in sediments in the Xianghu area (Table 3). A positive correlation was observed between the TOC contents and the clay composition (r = 0.55, *p* < 0.01), whereas a significant negative correlation was observed between the TOC contents and silt (r = −0.54, *p* < 0.01) in core XH-2 during 9300–6600 cal a BP. This indicates the TOC contents were high when the sediment particles were fine, whereas they declined when the sediment particles were coarse (Figure 5a,c,d). The reason may be that the surface adsorption of sediment particles played a significant role in the storage of organic matters. When the particles were coarse, the ability to store organic matters was weak, and TOC contents were low.

Studies demonstrated that sediment grain size is a vital indicator of the variability of hydrodynamic conditions. For example, the increase in coarse particulate matters reflects enhanced hydrodynamics, whereas the increase in fine particulate matters reflects a continuous weakening of hydrodynamics [27,37,38]. Organic carbon, representing the single largest constituent of organic matter, provides the most direct proxy for productivity [39,40,41,42]. Many studies of modern and ancient sediments have used TOC to reconstruct primary productivity [39,40,43,44,45,46]. Thus, TOC contents in core XH-2 can be used to indicate the hydrodynamics and primary productivity in the Xianghu area due to the superior correlation between TOC contents and grain size.

Additionally, Pearson correlation coefficients between δ^13^C and TOC in core XH-2 exhibited that the δ^13^C data presented a weak negative correlation (r = −0.37, *p* < 0.01) with TOC during the whole 9300–6600 cal a BP. However, the correlation between δ^13^C and TOC obviously varied from phase to phase. Specifically, they had moderate negative correlation (r = −0.44, *p* < 0.01) during 9300–8200 cal a BP, strong positive correlation (r = 0.53, *p* < 0.05) during 8200–7500 cal a BP, and remarkable negative correlation (r = −0.82, *p* < 0.01) during 7500–6700 cal a BP (Table 3). In general, the correlation between δ^13^C and TOC in core XH-2 was complex, which is also recorded in other studies [47,48,49,50]. The δ^13^C had a weak correlation with the proportion of clay and silt in sediments compared with TOC, suggesting that the δ^13^C was slightly influenced by grain size in core XH-2.

The TOC contents were stable and higher than the mean value during 9200–8200 cal a BP, with gradual increase in clay contents (Figure 5c), indicating that the hydrodynamic force in the study area was weak with relatively high productivity in the Xianghu area. This conformed the maximum diatom abundance in the Xianghu area during 9300–8200 cal a BP (Figure 5e). Therefore, a weak hydrodynamic force was conducive to diatom preservation, leading to high primary productivity in the Xianghu area.

The δ^13^C values during 9300–8200 cal a BP were mostly <−24‰ approximate to those of terrestrial C3 plants and freshwater algae [51], indicating that terrigenous organic matter and freshwater algae are possibly the major sources in the Xianghu area. This finding corresponds to the abundance of freshwater diatom during 9300–8200 cal a BP.

Additionally, a major influence of precipitation rates on the δ^13^C of land plant biomass has been documented for vegetation in areas with mean annual precipitation rates <2200 mm/year [51]. Other researchers hold relatively consistent opinions about the negative correlation between δ^13^C and precipitation. Reduced regional precipitation is associated with relatively high δ^13^C value due to reduction in isotopic fractionation during photosynthesis [37,52,53]. Thus, the δ^13^C data fluctuation in this study may be used to indicate precipitation changes in the Xianghu area.

The δ^13^C values of core XH-2 were relatively low during 9300–8200 cal a BP, indicating a humid climate in the Xianghu area, corresponding to a rapid sea-level rise in the Yangtze Delta [27]. The sample score on axis 1 exhibited a gradual increase, also suggesting a strong influence of sea water on the Xianghu area from 9000 to 8300 cal a BP (Figure 5f). This corresponded well with a gradual rise in sea level in the southern Hangzhou Bay [36]. Increase in marine diatom abundance in core XH-2 and core BMH [35,54] suggests that a rapid rise in sea level in the Xianghu area occurred between 8500 and 8300 cal a BP (Figure 4c). Additionally, a sharp increase in foraminifera during 8600–8400 cal a BP also indicates marine influences in the South Hangzhou Bay (core YJ1503) in association with a temporal rise in sea level in the region [27,55]. A combination of the geochemical data and diatom results infer the sedimentary environment underwent a transition from marsh or swamp to tidal deposit during 9300–8200 cal a BP.

### 5.2. 8200–7500 cal a BP

The TOC contents decreased during 8500–7500 cal a BP along with a slight decline in the composition of the clay contents, suggesting a slight increase in hydrodynamic force and declining primary productivity in the Xianghu area (Figure 5a,c). This finding was also inferred by a sharp decrease in diatom abundance since 8200 cal a BP [35] (Figure 5e).

The δ^13^C values during 8200–7500 cal a BP were relatively high (>24‰), indicating terrestrial C3 plants were the source of organic matter during this period, and suggesting an arid environment in the study area. An abrupt increase in δ^13^C at approximately 8200 cal a BP and the disappearance of diatom both in cores BMH and XH-2 indicated dry land setting in the study area (Figure 5b,e) [56]. Consequently, the Xianghu area was dried up and reduced to terrestrial environment and was then used as a settlement and farming place (Kuahuqiao culture 7700–7400 cal a BP).

Pollen records in the Kuahuqiao area also exhibited a cold and dry climate during 8200–7700 cal a BP [5,57,58,59,60,61]. On the basis of the pollen analysis of the Yuyao area in Zhejiang province, Li et al. [55] considered the ratio of *Arbors* in subtropical zone to those in warm temperate zone (warmness index, sub/tem) as a substitutive index for temperature to reconstruct the temperature change since Holocene. The results demonstrated that the Yuyao area was cold and dry during 8870–7500 cal a BP. The cold and dry climate during 8200–7500 cal a BP was also recorded by the pollen data at Yangtze River estuary [58]. This deduction was similar to our conclusion.

Pollen records of the Kuahuqiao site exhibited that the sedimentary environment in Kuahuqiao area was a reed swamp from 7700 cal a BP as human beings settled here and started planting rice [42,44]. The main sediment particles in core XH-2 during 7700–7500 cal a BP were silt and clay, the contents of which changed only slightly, indicating that the sedimental environment in the Xianghu area was stable, corresponding to the prosperity of the Kuahuqiao culture.

### 5.3. 7500–6600 cal a BP

The TOC contents stayed in a low-value level from 7400 to 6800 cal a BP (Figure 5a), corresponding to a marked decline in the clay composition and increase in the silt (Figure 5c,d), indicating relatively strong hydrodynamic force and low primary productivity. The disappearance of diatoms in the Xianghu area may be resulted from the strong hydrodynamic force, which was caused by both sea level rise and high precipitation during the Holocene optimum [60].

The δ^13^C values sharply dropped after approximately 7500 cal a BP (Figure 5b), suggesting a humid climate in the Xianghu area. Pollen records nearby the Kuahuqiao site also indicated that this area was influenced by the rising sea level since 7550 cal a BP when the site was submerged [20]. Shu et al. [5] and Innes et al. [59] reported that the Kuahuqiao area was influenced by transgression slightly later (7400 cal a BP). The TOC and δ^13^C values of core XH-2 suddenly dropped and tended to be partially negative in 7500 cal a BP, probably due to marine inundation [3]. During this episode, the Kuahuqiao culture ceased and eventually collapsed, attributed to the overwhelming marine transgression indicative of high sea level [3,59]. The marine transgression was well indicated at Xiasun, about 2 km north of Kuahuqiao, where abundant marine organisms were uncovered [5], and is also supported by the biostratigraphic study suggesting a high sea level in Hangzhou Bay in 7500 cal a BP [61].

## 6. Conclusions

The following preliminary conclusions were drawn from the TOC and δ^13^C in organic matters of sediments in the Xianghu area nearby Kuahuqiao site:

(1) Principal components analysis of diatom taxa exhibiting sample score on axis 1 can represent the strength of marine influence on the Xianghu area, suggesting a gradual increase in marine influence between 9000 and 8300 cal a BP in the study area.
(2) The TOC contents in core XH-2 were evidently influenced by grain size, but its correlation with δ^13^C was quite complex.
(3) Relatively high TOC contents and low δ^13^C values during 9300–8200 cal a BP reflected calmer waters and humid climate in the Xianghu area. During 8200–7500 cal a BP, a slight rise in TOC contents and remarkable increase in δ^13^C values indicated that the climate in the Xianghu area was arid. Diatom abundance decreased as the Xianghu area was gradually exposed, until becoming a land area. At approximately 7500 cal a BP, TOC contents and δ^13^C values declined, and the proportion of clay and silt also recorded obvious changes, suggesting the climate in the Xianghu area was humid accompanied by a relatively high sea level, and the Kuahuqiao site became obsolete. Thus, this study provides a basis for future work on Neolithic cultures in China.

## Figures and Tables

**Table 1:** Sample information for testing.

Test Index	Sample Information
Diatom	24 samples (with~100 cm sampling resolution)
Grain size	113 samples (with~20 cm sampling resolution)
TOC	113 samples (with~20 cm sampling resolution)
δ^13^C	113 samples (with~20 cm sampling resolution)

**Table 2:** AMS ^14^C ages of core XH-2 [35].

Laboratory Number	Depth (cm)	Dated Materials	^14^C Age (a BP)	Calibrated Age (cal a BP)
BETA430702	269	Plant fragment	5850 ± 30	6678.5 ± 63.5
BETA424911	475	Plant fragment	5950 ± 30	6745 ± 65
BETA416010	595	Organic-rich sediment	11,190 ± 40	13,060.5 ± 72.5
BETA430703	1253	Plant fragment	7470 ± 30	8319 ± 47
BETA416704	1851	Plant fragment	7950 ± 40	8815 ± 168
BETA416011	2520	Plant fragment	8320 ± 30	9349 ± 92

**Table 3:** Correlation coefficients between TOC contents, and grain size and δ^13^C, respectively.

Time Interval (cal a BP)	Clay (%)	Silt (%)	δ^13^C (‰)
9300–6600	0.55	−0.54	−0.37
9300–8200	−0.11 *	0.13 *	−0.44
8200–7500	0.61	−0.42	0.53
7500–6600	0.73	−0.71	−0.82

* The *p* value is greater than 0.05.

## Appendix A. Taxonomy

Based on the classification in Hasle and Syvertsen (1997) and Hendey (1964).

**Table A1:** Taxonomy.

ORDER	Coscinodiscales
**Family**	Hemidiscaceae
Genus	*Actinocyclus* Ehrenberg, 1837
	*Actinocyclus octonarius* Ehrenberg 1838
Genus	*Coscinodiscus* Ehrenberg, 1839
	*Coscinodiscus argus* Ehrenberg 1839
	*Coscinodiscus blandus* A.W.F. Schmidt 1878
	*Coscinodiscus ellipticus* Grunow, 1867 (1870)
	*Coscinodiscus lacustris* Grunow 1880
	*Coscinodiscus radiatus* Ehrenberg 1840
**Family**	Heliopeltaceae
Genus	*Actinoptychus senarius* Ehrenberg 1843
**ORDER**	Paraliales
**Family**	Paraliaceae
Genus	*Paralia* Heiberg, 1863
	*Paralia sulcata* (Ehrenberg) Cleve 1873
**ORDER**	Stephanodiscales
**Family**	Stephanodiscaceae
Genus	*Cyclotella* (Kützing) Brébisson, 1838
	*Cyclotella striata* Cleve and Grunow 1880
**ORDER**	Mastogloiales
**Family**	Achnanthaceae
Genus	*Achnanthes* Bory, 1822
	*Achnanthes minutissima* Kützing 1833
**ORDER**	Aulacoseirales
**Family**	Aulacoseiraceae
Genus	*Aulacoseira* Thwaites, 1848
	*Aulacoseira granulate* (Ehrenberg) Simonsen 1979
**ORDER**	Naviculales
**Family**	Diploneidaceae
Genus	*Diploneis* Ehrenberg ex Cleve, 1894
	*Diploneis smithii* (de brébisson) Cleve 1894
**ORDER**	Bacillariales
**Family**	Bacillariaceae
Genus	*Nitzschia* Hassall, 1845
	*Nitzschia granulata* Grunow 1880
	*Nitzschia levidensis* (W.Smith) Grunow 1881
**ORDER**	Thalassionematales
**Family**	Thalassionemataceae
Genus	*Thalassionema* (Grunow) Mereschkowsky 1902
	*Thalassionema nitzschioides* (Grunow) Mereschkowsky 1902
